# Rapid Detection of Free Cancer Cells in Intraoperative Peritoneal Lavage Using One-Step Nucleic Acid Amplification (OSNA) in Gastric Cancer Patients

**DOI:** 10.3390/cells9102168

**Published:** 2020-09-25

**Authors:** Katarzyna Gęca, Karol Rawicz-Pruszyński, Jerzy Mielko, Radosław Mlak, Katarzyna Sędłak, Wojciech P. Polkowski

**Affiliations:** 1 Department of Surgical Oncology, Medical University of Lublin, Radziwiłłowska 13 St., 20-080 Lublin, Poland; kasiaa.geca@gmail.com (K.G.); jerzy.mielko@umlub.pl (J.M.); sedlak.katarz@gmail.com (K.S.); wojciech.polkowski@umlub.pl (W.P.P.); 2 Department of Human Physiology, Medical University of Lublin, Radziwiłłowska 11 St., 20-080 Lublin, Poland; radoslaw.mlak@gmail.com

**Keywords:** gastric cancer, One-Step Nucleic Acid amplification, free cancer cells, peritoneal fluid, peritoneal lavage

## Abstract

Cytokeratin-19 (CK19) has been proven to be commonly expressed by cancer cells in a variety of solid tumors and may serve as a suitable marker of metastases in gastric cancer (GC). Since objective assessment of peritoneal lavage or fluid for free cancer cells (FCC) is essential for clinical decision making in patients with GC, it is important to develop a quantitative and reproducible method for such evaluation. We assessed the possible application of One-Step Nucleic Acid amplification (OSNA) assay as a rapid method for FCC detection in intraoperative peritoneal lavage or fluid of GC patients. Seventy-eight intraoperative peritoneal lavage or fluid samples were eligible for the analysis by conventional cytology and OSNA examination. The concentration of CK19 mRNA in intraoperative peritoneal lavage and fluid was compared with the conventional cytological assessment. CK19 mRNA concentration was detected by OSNA assay. For peritoneal lavage samples, sensitivity and specificity were 83.3% and 87.8%, respectively. In peritoneal fluid, significantly higher CK19 values were observed in patients with serosal infiltration (medians: 100 copies/µL vs. 415.7 copies/µL; *p* = 0.0335) and lymph node metastases (medians: 2.48 copies/µL vs. 334.8 copies/µL). OSNA assay turns out to be an objective, fast, and reproducible quantitative method of FCC assessment.

## 1. Introduction

Peritoneal dissemination is a common cause of relapse in gastric cancer (GC) patients and is related to poor prognosis with median survival of approximately one year [1,2,3,4,5,6]. It is considered that peritoneal spread is most likely caused by free cancer cells (FCC), which exfoliated from primary gastric tumor or involved lymph nodes. According to the TNM classification, the presence of FCC in peritoneal fluid is considered as distant metastasis (CY1) and qualified as stage IV of disease [1,2,3,4].

Patients with peritoneal lavage positive by cytology and without macroscopic peritoneal dissemination have similar survival rates to the patients with overt peritoneal metastasis. The five-year survival rate of such patients in gastric cancer is only 2% with median survival of 9.2 months [7]. The FCC are able to implant into peritoneal surface and metastasize [2]. The presence of FCC in peritoneal fluid has been used as a prognostic and predictive factor of peritoneal recurrence and overall survival [3]. Moreover, approximately 17% of patients undergoing resection with curative intent will develop peritoneal spread, which is associated with poor outcome [3].

Among numerous methods for detecting FCC in peritoneal fluid, cytological evaluation after hematoxylin and eosin (H&E) or Papanicolau staining is considered a gold standard [5]. However, the usefulness of conventional cytology for prediction of peritoneal metastasis is controversial because of its low sensitivity, ranging from 11 to 80% [1,6]. Differences in sensitivity may be caused by the fact that conventional cytology is based only on qualitative morphological examination, which could be challenging even for experienced pathologist. Conventional qualitative cytology has low sensitivity, and is largely dependent on the institution as well as the pathologist, which sometimes results in confusion in the clinical evaluation of the outcome of cytology-positive patients [5]. Immunohistochemistry (IHC) with a panel of monoclonal antibodies directed to GC associated antigens help to increase the detection rate of the FCC up to 15% when compared to conventional cytology [1]. Molecular diagnostics using RT-PCR also has been used to detect FCC in peritoneal fluid due to its high sensitivity [1,5]. Usefulness of molecular techniques for detecting tumor markers, such as carcinoembryonic antigen (CEA), cytokeratin 19 (CK-19), and cytokeratin 20 (CK-20), have been reported [1,5].

Recently, an automated molecular diagnostic assay has been developed to detect lymph node metastasis in breast, colorectal, gastric, lung, endometrial, cervical, and prostate cancer patients [1,8,9,10,11,12,13]. The One-Step Nucleic acid Amplification (OSNA) method consists of easy sample preparation and detection of targeted CK-19 mRNA sequences using reverse transcription-loop mediated isothermal amplification (RT-LAMP) technique [8,9,14]. The RT-LAMP technique amplifies a targeted mRNA with high specificity, efficiency, and rapidity under isothermal conditions [8,9,14,15]. Amplification of targeted gene is detectable in real-time by an increase of turbidity of the solution derived from a side product of the reaction, which can be completed within 30 min [14,15]. The expression level of CK-19 mRNA correlates with the size of metastatic foci [14].

Since objective assessment of peritoneal lavage or fluid for Free Cancer Cells (FCC) is essential for clinical decision making in patients with GC, it is important to develop a quantitative and reproducible method for such evaluation. The aim of our study was to verify the usefulness of OSNA assay to detect the FCC in intraoperative peritoneal lavage or fluid from GC patients.

## 2. Materials and Methods

**Figure 1:**
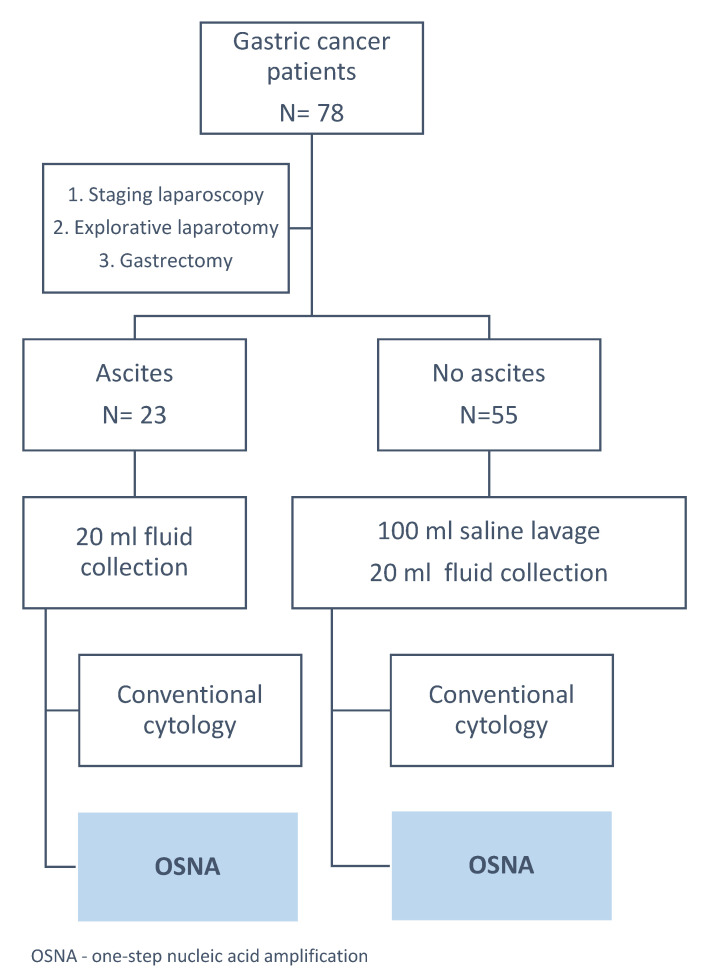
Study design. OSNA: One-Step Nucleic Acid Amplification.

The study was conducted after obtaining institutional review board approval (Bioethical Committee of Medical University of Lublin, Ethic Code: Ke–0254/297/2018). We collected data from a prospectively maintained database of all patients operated on GC between August 2018 and March 2020 in the Department of Surgical Oncology, Medical University of Lublin, Poland. In total, 78 peritoneal washes samples were eligible for the analysis. A flow chart of the study is presented in Figure 1.

### 2.1. Intraoperative Peritoneal Lavage and Fluid Examination

Peritoneal fluid was collected during: 1. diagnostic laparoscopy, 2. exploratory laparotomy, 3. surgery. In case of an absence of abdominal ascites, lavage cytology was performed. Abdominal cavity was washed with 100 mL of physiological saline solution and gently stirred. Then, peritoneal lavage was aspirated from the tumor area. The washings or fluid obtained were divided into two equal parts. One was intended for cytological examination, while the other one was centrifuged for 10 min at 1500× *g* in room temperature in order to obtain cellular sediment. The cell pellet obtained in this way was stored in −80 °C, until the time of examination with OSNA, performed on the postoperative day 1.

### 2.2. OSNA Examination

The protocol for OSNA performance has been slightly modified to adapt it to peritoneal fluid examination. The first step of sample preparation was homogenization of cellular sediment, which was carried out with a Fast Prep device (MP Biomedicals, Irvine, CA, USA). One mL of homogenizing buffer LYNORHAG, pH 3.5 (Sysmex, Kobe, Japan) was added to cellular pellet. The cells with homogenizing buffer were transferred to Fast Prep lysing matrix tubes and homogenized for 2 min at 6.5 m/s. First, 600 µL of homogenate was centrifuged for 1 min at 1000× *g*, followed by 500 µL of OSNA Lysate transfer to the microtube. Then, 100 µL of this lysate was placed in spin columns and centrifuged for 2 min at 375× *g*. Twenty µL of centrifuged OSNA Lysate was then transferred to a vial tube with pre-set 180 µL of LYNORHAG for analysis. RT-LAMP reaction was accomplished with a ready-to-use LYNOAMP gene amplification reagent kit (Sysmex, Kobe, Japan) on the RD-100i gene amplification detector (Sysmex) [14]. Reaction mixture contained tree pairs of primers: 5′-GGAGTTCTCAATGGTGGCACCAACTACTACACGACCATCCA-3′ (CK19 forward inner primer), 5′-GTCCTGCAGATCGACAACGCCTCCGTCTCAAACTTGGTTCG-3′ (CK19 reverse inner primer), 5′-TGGTACCAGAAGCAGGGG-3′ (CK19 forward outer primer), 5′-GTTGATGTCGGCCTCCACG-3′ (CK19 reverse outer primer), 5′-AGAATCTTGTCCCGCAGG-3′ (CK19 forward loop primer), and 5′-CGTCTGGCTGCAGATGA-3′ (CK19 reverse loop primer) [16]. The RT-LAMP method measured the time taken to exceed a predetermined threshold turbidity caused by magnesium pyrophosphate, which is a by-product of the amplification reaction [17]. The change in turbidity correlates with the CK19 mRNA copy number calculated from the value of the standard curve, which was previously determined with three calibrators containing different concentration of CK19 mRNA copy number/µL [14,18]. Limit of detection (LOD) of the RD-100i gene amplification detector was set at 250 copy number/µL. The results below the LOD were calculated based on the standard curve.

### 2.3. Statistical Analysis

MedCalc software v. 15.8 was used for statistical analysis. Discontinuous (categorized) data are presented as percentages. The normality of the distribution of continuous data was assessed using the D’Agostino-Pearson test. Since compared data had non-normal distribution, non-parametric tests were used. Comparisons were performed using U-Mann Whitney (comparisons between two groups) or Kruskal-Wallis (comparison between more than two groups) tests. Assessment of OSNA diagnostic utility in FCC detection in tested materials was performed with the use of ROC analysis. In all cases, *p*-value < 0.05 was considered as statistically significant.

## 3. Results

Among 78 samples included in the study, 43 (55.1%) were taken from males and 35 (44.9%) from females. The median age of patients was 63 years. The examined material consisted of 55 (70.5%) intraoperative peritoneal lavage and 23 (29.5%) peritoneal fluid samples. According to Lauren histological classification, there were 29 (37.2%) intestinal, 11 (14.1%) mixed, and 34 (43.6%) diffuse-type tumors. Four (7.7%) of cases were not classified. Over half of the patients included in the study (55.6%) were at least pT3. Additionally, 22 (48.7%) patients had lymph nodes metastases (pN+) and six (14.6%) patients had distant metastases (pM1). In peritoneal fluid and intraoperative peritoneal lavage samples, positive cytology (c+) was found in 13 (56.5%) and six (10.9%) patients, respectively. The clinicopathological features of selected patients are shown in Table 1.

### 3.1. Comparison of CK19 Levels in Selected Clinical Variables

Significantly higher CK19 values were found in the peritoneal fluid compared to intraoperative peritoneal lavage (medians: 200 copies/µL vs. 0 copies/µL; *p* = 0.0002). In peritoneal fluid, significantly higher CK19 values were observed in patients with serosal infiltration (medians: 100 copies/µL vs. 415.7 copies/µL; *p* = 0.0335) and lymph node metastases (medians: 2.48 copies/µL vs. 334.8 copies/µL; *p* = 0.0478). In positive cytology, significantly higher values of CK19 were observed in intraoperative peritoneal lavage (medians: 577.5 copies/µL vs. 0 copies/µL; *p* = 0.0027) and peritoneal fluid (medians: 960 copies/µL vs. 6.9 copies/µL; *p* = 0.0099). Detailed data on CK19 expression in selected clinical variables are presented in Table 2.

### 3.2. OSNA Assay in Detecting FCC in Intraoperative Peritoneal Lavage and Peritoneal Fluid with Positive Cytology Using ROC Curve Analysis

**Figure 2:**
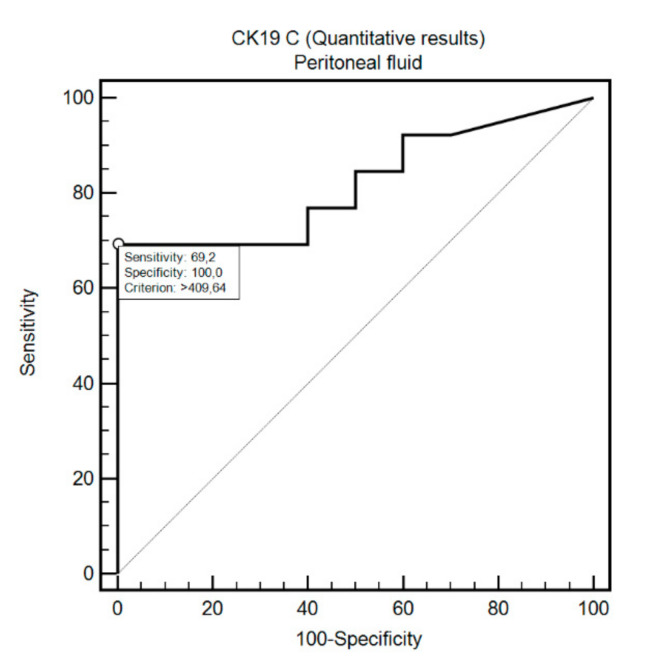
Diagnostic usefulness of CK19 concentration in differentiating positive and negative cytology in the peritoneal fluid samples.

**Figure 3:**
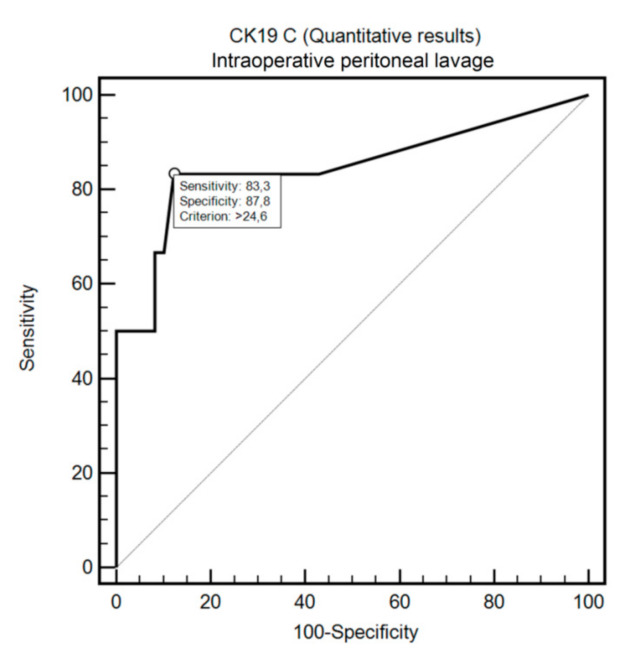
Diagnostic usefulness of CK19 concentration in differentiating positive and negative cytology in the intraoperative peritoneal lavage.

OSNA assay detected FCC in the peritoneal fluid with positive cytology with 69.2% sensitivity and 100% specificity (criterion: >409.64; AUC = 0.82, 95%CI: 0.60–0.95, *p* = 0.0005; Figure 2). Similarly, OSNA assay detected FCC in the intraoperative peritoneal lavage with positive cytology with 83.3% sensitivity and 87.8% specificity (criterion: >24.6; AUC = 0.85, 95%CI: 0.73–0.93, *p* = 0.0025; Figure 3).

## 4. Discussion

The present study evaluated the potential role of the OSNA assay in detecting FCC in intraoperative peritoneal lavage and fluid in patients with GC. OSNA could successfully identify FCC in both evaluated materials. When compared with conventional peritoneal cytology in GC patients, OSNA assay is characterized by rapid (30 min), objective, quantitative, and reproducible assessment.

Similarly to our study, Nakabayashi et al. [1] evaluated OSNA for detecting FCC in peritoneal washings from GC patients. However, instead of CK19, CEA mRNA was used as a cancer cells marker. A cut-off value to distinguish between cytologically positive and negative results for OSNA mRNA using the ROC curve analysis was set, and the concordance rate between both methods was 93.8%. The sensitivity and specificity were 85.0% and 97.7%, respectively, which is similar to our findings in intraoperative peritoneal lavage group (83.3% and 87.8%, respectively). Yoneda et al. [19] measured the expression of CK19 mRNA in intraoperative peritoneal lavage in 52 GC patients using the RT-LAMP technique. A significant correlation between CK19 mRNA levels and recurrence was found. RT-LAMP-positive patients had worse outcome than RT-LAMP-negative patients. The RT-LAMP technique additionally correlated with tumor depth and lymph node metastases, analogously to our study.

Nevertheless, amplified mRNA may originate from dead cells or phagocytes that have absorbed tumor cells, and can be released from hematopoietic and inflammatory cells [20]. Therefore, the clinical issue of false positive cases remains to be resolved. On the other hand, the use of mRNA markers is founded on the assumption that cancer cells still present the same expression of the antigen as their tissue of origin. After the separation from cancer cells, mRNAs are fairly unstable. For this reason, detection of mRNA markers indicate the presence of viable neoplastic cells in the examined material [21]. CK19 has been proven to be commonly expressed by cancer cells of epithelial origin but not by lymphoid or hematopoietic cells [22]. Majima et al. [23] reported that CK19 may exceed CK20 in detecting circulating cancer cells in peripheral blood by means of RT-PCR. That is why CK19 may serve as a suitable marker of metastases in GC patients.

For GC patients with positive cytology, but without gross peritoneal metastases, no clear therapeutic strategies have yet been established. Studies conducted by Hyun-Jeong et al. [24] have shown that postoperative chemotherapy improves the survival outcome compared to surgery alone in GC patients who underwent radical D2 gastrectomy. Since the presence of FCC in the peritoneal fluid/lavage influences further therapeutic decisions, it is important that the test result is reliable.

OSNA can be performed as an alternative to an intraoperative cytological examination. Due to its high sensitivity and speed of operation, this method could provide an opportunity to perform an accurate, tailor-made surgical procedure. Even though the exact mechanism of peritoneal spread is still unclear, the presence of cancer cells during surgery may lead to a relapse in the peritoneum [2]. Recently, the use of Extensive Intraoperative Peritoneal Lavage method (EIPL) has been in a high demand as a new prophylactic strategy for treatment of peritoneal metastasis of locally advanced GC [25]. Currently, three multicenter randomized clinical trials are conducted to assess the safety and efficacy of EIPL. The short-term outcomes of the SEIPLUS trial have been published. It has been proven that EIPL can increase the safety of D2 gastrectomy and decrease postoperative short-term complications and wound pain [25]. On the other hand, Misawa et al. [26] in their open-label, multi-institutional, randomized, phase 3 trial (CCOG 1102 trial), reported that EIPL did not improve survival or peritoneal recurrence in patients who underwent gastrectomy for advanced GC. The OSNA system seems to be an ideal tool for monitoring the effectiveness of EIPL. Thanks to its rapidity, the results can be available intraoperatively. This study contains certain limitations. Due to its retrospective nature, it cannot identify causation. Additionally, we did not assess the peritoneal fluid/lavage with other molecular biological techniques, such as quantitative reverse-transcription PCR (QRT-PCR). A combined assay (OSNA and QRT-PCR) in the future studies may result in an increased clinical utility of FCC assessment in GC patients. Moreover, due to the relatively small sample size and heterogeneity of the study, our results should be confronted with further, large scale studies.

## 5. Conclusions

OSNA assay detects FCC in intraoperative peritoneal lavage and fluid in GC patients with high sensitivity and specificity. When compared with conventional peritoneal cytology in GC patients, OSNA assay turns out to be an objective, fast, and reproducible quantitative method of FCC assessment in the peritoneal cavity.

## Figures and Tables

**Table 1:** Clinicopathological variables.

Variable	Peritoneal Fluid	Intraoperative Peritoneal Lavage
	n (%) or Median ± SD. Median (Range)	
Sex		
Males	11 (47.8%)	32 (58.2%)
Females	12 (52.2%)	23 (41.8%)
Age	60.2 + 12.6.	62.4 + 11.4
	60 (36–86)	63 (37–87)
Group		
Explorative laparotomy (M1)	12 (52.2%)	6 (10.9%)
Staging Laparoscopy	3 (13.0%)	16 (29.1%)
Surgery after CTH	7 (30.4%)	25 (45.4%)
Upfront Surgery	1 (4.4%)	8 (14.5%)
Lauren type		
Intestinal	4 (17.4%)	25 (45.5%)
Mixed	6 (26.1%)	5 (9.1%)
Diffused	12 (52.2%)	22 (40.0%)
Unclassified	1 (4.3%)	3 (5.4%)
pT		
1a	1 (10%)	6 (19.3%)
1b	1 (10%)	3 (9.7%)
2	2 (20%)	4 (12.9%)
3	2 (20%)	10 (32.3%)
4a	1 (10%)	6 (19.3%)
4b	3 (30%)	2 (6.4%)
pN		
0	4 (40%)	17 (54.8%)
1	1 (10%)	5 (16.1%)
2	1 (10%)	4 (12.9%)
3, 3a, 3b	4 (40%)	5 (16.1%)
pM		
0	7 (70%)	28 (90.3%)
1	3 (30%)	3 (9.7%)
Cytology		
Positive	13 (56.5%)	6 (10.9%)
Negative	10 (43.5%)	49 (89.1%)

pT: pathologic tumor status, pN: pathologic node status, pM: pathologic metastasis status.

**Table 2:** Comparison of Cytokeratin-19 (CK19) concentration values depending on selected clinical variables.

Variable	Peritoneal Fluid		Intraoperative Peritoneal Lavage	
	Median (copies/µL)	*p*	Median (copies/µL)	*p*
pT		0.0335		0.6778
1a/b, 2	2.48		0	
3	100		0.03	
4a, 4b	415.7		0	
pN		0.0478		0.6264
0	2.48		0	
1–3	334.8		0	
pM		0.1056		0.0125
0 (C−)	5		0	
0 (C+)	38508.2		2200	
1 (C−)	25.1		0	
1 (C+)	960		54.9	
Cytology		0.0099		0.0027
Positive	960		577.5	
Negative	6.9		0	

C−: negative cytology, C+: positive cytology.
